# Ultrasensitivity in signaling cascades revisited: Linking local and global ultrasensitivity estimations

**DOI:** 10.1371/journal.pone.0180083

**Published:** 2017-06-29

**Authors:** Edgar Altszyler, Alejandra C. Ventura, Alejandro Colman-Lerner, Ariel Chernomoretz

**Affiliations:** 1 Laboratorio de Inteligencia Artificial Aplicada, Universidad de Buenos Aires, Departamento de Computación - CONICET, Ciudad Universitaria, Pabellón I, Buenos Aires, C1428EHA, Argentina; 2 IFIBYNE-UBA-CONICET and Departamento de Fisiología, Biología Molecular y Celular, Facultad de Ciencias Exactas y Naturales, Universidad de Buenos Aires, Ciudad Universitaria, Pabellón II, Buenos Aires, C1428EHA, Argentina; 3 Departamento de Física FCEN UBA - IFIBA CONICET, Ciudad Universitaria, Pabellón I, Buenos Aires, C1428EHA, Argentina; 4 Fundación Instituto Leloir, Av Patricias Argentinas 435, C1405BWE, Buenos Aires, Argentina; University of PECS Medical School, HUNGARY

## Abstract

Ultrasensitive response motifs, capable of converting graded stimuli into binary responses, are well-conserved in signal transduction networks. Although it has been shown that a cascade arrangement of multiple ultrasensitive modules can enhance the system’s ultrasensitivity, how a given combination of layers affects a cascade’s ultrasensitivity remains an open question for the general case. Here, we introduce a methodology that allows us to determine the presence of sequestration effects and to quantify the relative contribution of each module to the overall cascade’s ultrasensitivity. The proposed analysis framework provides a natural link between global and local ultrasensitivity descriptors and it is particularly well-suited to characterize and understand mathematical models used to study real biological systems. As a case study, we have considered three mathematical models introduced by O’Shaughnessy et al. to study a tunable synthetic MAPK cascade, and we show how our methodology can help modelers better understand alternative models.

## Introduction

Sigmoidal input-output response modules are well-conserved in cell signaling networks. They might be used to implement binary responses, a key element in cellular decision-making processes. Additionally, sigmoidal modules might be part of more complex structures, where they can provide the nonlinearities which are needed in a broad spectrum of biological processes [1, 2], such as multistability [3, 4], adaptation [5], and oscillations [6]. There are several molecular mechanisms that are able to produce sigmoidal responses, such as inhibition by titration [7, 8], zero-order ultrasensitivity in covalent cycles [9, 10], and multistep activation processes such as multisite phosphorylation [11–15] or ligand binding to multimeric receptors [16].

Sigmoidal curves are characterized by a sharp transition from low to high output following a slight change in the input. The steepness of this transition is called ultrasensitivity [10]. In general, the following operational definition of the Hill coefficient may be used to calculate the overall ultrasensitivity of sigmoidal modules:

$$\begin{array}{c} n_{H}=\frac{\log(81)}{\log(EC90/EC10)} \end{array}$$ (1)

where EC10 and EC90 are the signal values needed to produce an output of 10% and 90% of the maximal response, respectively. These two values delimit the input dynamic range. The Hill coefficient *n*_*H*_ quantifies the steepness of a transfer function relative to the hyperbolic response function which is defined as not ultrasensitive and has *n*_*H*_ = 1. An *n*_*H*_ = 1 means that an 81-fold increase in the input signal is required to change the output level from 10% to 90% of its maximal value. Response functions with *n*_*H*_ > 1 need a smaller input fold increase to produce such output change, and are called ultrasensitive functions.

Global sensitivity measures, such as the one described by Eq 1 do not fully characterize sigmoidal curves, y(x), because they average out local characteristics of the analyzed response functions. Instead, these local features are well captured by the *logarithmic gain* or *response coefficient* [17] defined as:

$$\begin{array}{c} R\left(x\right)=\frac{x}{y}\frac{dy}{dx}=\frac{d\log(y)}{d\log(x)} \end{array}$$ (2)

Eq 2 provides local ultrasensitivity estimates given by the local polynomial order of the response function.

### Mitogen activated protein kinase (MAPK) cascades

MAPK cascades are well-conserved. They can be found in a broad variety of cell fate decision systems involving processes such as proliferation, differentiation, survival, development, stress response and apoptosis [18]. They are composed of a chain of three kinases which sequentially activate one another, through single or multiple phosphorylation events. An experimental and mathematical study of this kind of systems was performed by Ferrell and collaborators, who analyzed the steady-state response of a MAPK cascade that operates during the maturation of oocytes in *Xenopus laevis* [19]. They developed a biochemical model to study the ultrasensitivity at each of the cascade’s layers and reported that the combination of ultrasensitive layers into a multilayer structure enhanced the overall system’s ultrasensitivity [19]. Similary, Brown et al. [20] showed that if the dose-response curve of a cascade, *F*(*x*), could be described as the mathematical composition of functions, *f*^*is*^, each of which describes the behavior of each layer in isolation (i.e, $F\left(x\right)=f_{MK}^{is}\left(f_{MKK}^{is}\left(f_{MKKK}^{is}\left(x\right)\right)\right)$, then the local ultrasensitivity of the different layers combines multiplicatively:

$R\left(x\right)=R_{MK}(f_{MKK}^{is}\left(f_{MKKK}^{is}\left(x\right)\right).R_{MKK}\left(f_{MKKK}^{is}\left(x\right)\right).R_{MKKK}\left(x\right)$.

In connection with this result, Ferrell showed, for the special case of two Hill-type modules of the form

$$\begin{array}{c} y=k\frac{x^{n_{H}}}{{EC50}^{n_{H}}+x^{n_{H}}} \end{array}$$ (3)

(where the parameter EC50 corresponds to the value of input that elicits half-maximal output, and *n*_*H*_ is the Hill coefficient), that the overall cascade global ultrasensitivity had to be less than or equal to the product of the global ultrasensitivity estimators of each cascade’s layer, i.e *n*_*H*_ ≤ *n*_*H*,1_ *n*_*H*,2_ [13].

Hill functions of the form given by Eq 3 are normally used as phenomenological approximations of sigmoidal dose-response curves, even without any mechanistic basis [2]. However, the composition of sigmoidal transfer functions, different from Hill-functions, may not satisfy Ferrell’s inequality. In particular, a supra-multiplicative behavior (the ultrasensitivity of the combination of layers is higher than the product of each of the layer’s ultrasensitivity) might be observed for left-ultrasensitive response functions, i.e. functions that are steeper to the left of the EC50 than to the right. In this case, the boost in the ultrasensitivity is caused by the asymmetrical dose-response functional form (see [21] for details).

Since modules are usually embedded in larger networks, the actual input dynamic range of the module could be constrained. We recently formalized this idea introducing the concept of *dynamic range constraint of a module’s dose-response function*. It is a feature inherently linked to *cascading* (coupling of modules in a multilayer architecture), and its consideration helps explain the overall ultrasensitivity displayed by a given cascade [21]. Besides dynamic range constraint effects, sequestration (i.e. the reduction in free active enzyme due to its accumulation in complexes with its substrates) is another relevant process inherent to cascading. In this case, sequestration leads to a reduction in the cascade’s ultrasensitivity [22–24]. Moreover, sequestration may alter the qualitative features of any well-characterized module when integrated with upstream and downstream components, thereby limiting the validity of module-based descriptions [25–27].

All these considerations qualify the concept of modularity, which requires the isolation of a particular processing units (or module), and highlight the importance of studying their behavior when embedded in large networks. Although there has been significant progress in the understanding of kinase cascades, how the combination of layers affects the cascade’s ultrasensitivity remains an open question for the general case.

In the present work, we have developed a method to describe the overall ultrasensitivity of a kinase cascade in terms of the effective contribution of each module. We used our approach to analyze a recently presented synthetic MAPK cascade experimentally engineered by O’Shaughnessy et al. [28].

O’Shaughnessy et al. [28] expressed a mammalian MAPK cascade (a Raf-MEK-ERK system) in the yeast *S. cerevisiae* modified so as to be able to activate Raf with the hormone estradiol. In this setup, they measured input output dose responses and made use of a mechanistic mathematical to help understand the results. Their model was very similar in spirit to the Huang-Ferrell model [19] with two important differences: a) they did not include phosphatases, and b) they explicitly modeled synthesis and degradation of all species. Interestingly, they reported that the multilayer structure of the analyzed cascades accumulated ultrasensitivity supramultiplicatively, and suggested that a cascading effect and not any other process (such as multi-step phosphorylation, or zero-order ultrasensitivity) was responsible for the supramultiplicative behavior. They called this mechanism *de-novo ultrasensitivity generation*. We found the proposed mechanism unexpected and thus we wanted to characterize it within our analysis framework.

The paper is organized as follows. First, we present a formal connection between local and global descriptors of a module’s ultrasensitivity for the case of a cascade composed of *N* units. We then introduce the notion of Hill input’s working range in order to analyze how a module embedded in a cascade contributes to the overall system’s ultrasensitivity. Next, we describe a simple methodology to identify the presence of sequestration effects that might affect the system ultrasensitive behavior. Finally, as a case study, we present the O’Shaughnessy cascade analysis. We conclude by presenting a summarizing discussion and the conclusions of the work.

## Results

### Linking local and global ultrasensitivity estimations

The concept of ultrasensitivity describes a module’s ability to amplify small changes in input values into larger changes in output values. It is customary to quantify and characterize the extent of the amplification both globally, using the Hill coefficient *n*_*H*_ defined in Eq 1, and locally, using the response coefficient, R(I), as a function of the module’s input signal I (Eq 2), We found a simple relationship between both descriptions considering the logarithmic amplification coefficient $A_{a,b}^{f}$, defined as:

$$\begin{array}{c} A_{a,b}^{f}=\frac{\log(f(b))-\log(f(a))}{\log(b)-\log(a)} \end{array}$$ (4)

**Fig 1:**
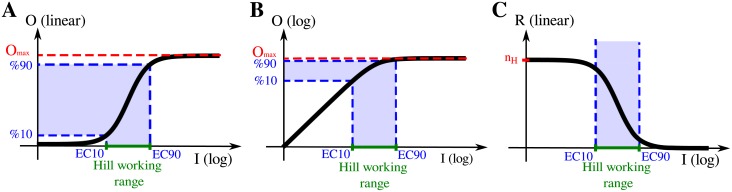
Hill function dose-response. Schematic representation of Hill-type dose-response curves, in log-linear (A) and log-log scale (B). The EC10 and EC90 are the inputs needed to produce an output of 10% and 90% of the maximal response (*O*_*max*_), respectively. The *Hill working range*, HWR, is the input range relevant for the calculation of the system’s *n*_*H*_. For isolated modules, the HWR = [EC10, EC90]. Panel (C) displays the local ultrasensitivity (the response coefficient R) as a function of input. Note that for Hill functions, inputs much smaller than the EC50 have Rs around the Hill coefficient.

$A_{a,b}^{f}$ describes the change (in a logarithmic scale) produced in the output when the input varies from a to b. For instance, $A_{a,b}^{f}=0.5$ for an hyperbolic function evaluated between the inputs that result in 90% and 10% of the maximal output. In this case, the two inputs considered delimit the input range that is relevant for the estimation of the Hill coefficient *n*_*H*_. We call this input interval the *Hill working range* (HWR) (see Fig 1A and 1B).

Taking into account Eq 4, the parameter *n*_*H*_ can be rewritten as follows,

$$\begin{array}{c} n_{H}=\frac{\log(81)}{\log(EC90/EC10)}=\frac{2\log(0.9/0.1)}{\log(EC90/EC10)}=2A_{EC10,EC90}^{f}=\frac{A_{EC10,EC90}^{f}}{A_{EC10,EC90}^{hyp}} \end{array}$$ (5)

Consequently, the Hill coefficient may be interpreted as the ratio of the logarithmic amplification coefficients of the function of interest and an hyperbolic function, evaluated in the corresponding HWR.

It is worth noting that the logarithmic amplification coefficient that appears in Eq 5 equals the slope of the line that passes through the points (*EC*10, *f*(*EC*10)) and (*EC*90, *f*(*EC*90)) in a log-log scale. Thus, this quantity equals the average response coefficient calculated over the interval of the *HWR* = [*EC*10, *EC*90], in logarithmic scale (see Fig 1C). If follows that

$$\begin{array}{c} n_{H}=2A_{EC10,EC90}^{f}=2\frac{\int_{\log(EC10)}^{\log(EC90)}R_{f}\left(I\right)d\left(\log I\right)}{\log(EC90)-\log(EC10)}=2{\left\langle R_{f}\right\rangle }_{EC10,EC90}=\frac{{\left\langle R_{f}\right\rangle }_{EC10,EC90}}{{\left\langle R_{hyp}\right\rangle }_{EC10,EC90}} \end{array}$$ (6)

where 〈*X*〉_*a*, *b*_ denotes the mean value of the variable x over the range [a, b].

This last equation explicitly links the local and global ultrasensitivity descriptions.

### Ultrasensitivity in function composition

Next, we generalized the above result to express the overall global ultrasensitivity of a multilayer cascade in terms of logarithmic amplification coefficients. We first considered two coupled ultrasensitive modules, disregarding effects of sequestration of molecular components between layers. In this case, the expression for the system’s dose-response curve, *F*, results from the mathematical composition of the functions, *f*_*i*_, each of which which describes the input/output relationship of isolated modules *i* = 1, 2:

$$\begin{array}{c} F\left(I_{1}\right)=f_{2}(f_{1}\left(I_{1}\right)) \end{array}$$ (7)

**Fig 2:**
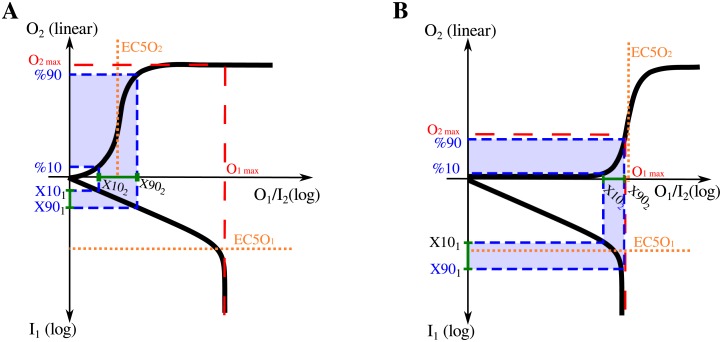
Hill functions composition. Schematic response function diagrams for two different compositions of a pair of Hill-type ultrasensitive modules. In each panel, the dose-response function of the first module is displayed in the lower semi-plane: the downward vertical axis representing the first module’s input signal while its response function, which corresponds to the second module’s input, is displayed along the horizontal axis. The dose-response curve for the second module is displayed in the upper-plane. In (A) the maximum output of the first module is higher than the EC50 of the second module (*O*_1,*max*_ ≫ *EC*50_2_), while in (B), it is lower than that value (*O*_1,*max*_ < *EC*50_2_).

Using Eq 5:

$$\begin{array}{ccc} n_{H} & = & \frac{\log(81)}{\log(X90_{1}/X10_{1})}=2\overset{\nu_{2}}{\overset{︷}{\frac{\log(0.9/0.1)}{\log(X90_{2}/X10_{2})}}}\overset{\nu_{1}}{\overset{︷}{\frac{\log(X90_{2}/X10_{2})}{\log(X90_{1}/X10_{1})}}}\\ & = & 2\overset{\nu_{2}}{\overset{︷}{A_{X10_{2},X90_{2}}^{f_{2}}}}\overset{\nu_{1}}{\overset{︷}{A_{X10_{1},X90_{1}}^{f_{1}}}}=2\overset{\nu_{2}}{\overset{︷}{{\left\langle R_{2}\right\rangle }_{X10_{2},X90_{2}}}}\overset{\nu_{1}}{\overset{︷}{{\left\langle R_{1}\right\rangle }_{X10_{1},X90_{1}}}}\\ & = & 2\;\nu_{2}\;\nu_{1} \end{array}$$ (8)

where *X*10_*i*_ and *X*90_*i*_ are the boundaries of the HWR of the composite system, i.e. the input values for the i-layer that produce a 10% and 90% of the system’s maximal response, respectively (see Fig 2).

When composing two functions, there are two extreme scenarios. In the first, the maximum output level of the first module may exceed by far the EC50 of the second module: *O*_1,*max*_ ≫ *EC*50_2_ (Fig 2A). In this case *O*_2,*max*_ equals the maximum output level of module 2 in isolation, *X*10_2_ and *X*90_2_ match the EC10 and EC90 levels of module 2 in isolation and the HWR of module 1 is located in the input region below *EC*50_1_ (Fig 2A). In the second scenario, the maximum output of the first module may be lower than the EC50 of the second module: *O*_1,*max*_ < *EC*50_2_ (Fig 2B). Here, the HWR [*X*10_2_, *X*90_2_] is shifted with respect to the input range [EC10, EC90] that would have been considered for module 2, if analyzed in isolation. As a result, module-1’s HWR is centered at values higher than the corresponding EC50 level.

It follows from Eq 8 that the system’s Hill coefficient *n*_*H*_ depends on the product of two factors, *ν*_1_ and *ν*_2_, which characterize local average sensitivities over the relevant input region for each layer: [*X*10_*i*_, *X*90_*i*_], with *i* = 1, 2 (see Fig 2). We call coefficient *ν*_*i*_ the *effective response coefficient of layer-i*.

For the more general case of a cascade of *N* modules we found that:

$$\begin{array}{c} n_{H}=2\overset{\nu_{N}}{\overset{︷}{{\left\langle R_{N}\right\rangle }_{X10_{N},X90_{N}}}}\overset{\nu_{N-1}}{\overset{︷}{{\left\langle R_{N-1}\right\rangle }_{X10_{N-1},X90_{N-1}}}}\ldots .\overset{\nu_{1}}{\overset{︷}{{\left\langle R_{1}\right\rangle }_{X10_{1},X90_{1}}}}=2\;\nu_{N}\;\nu_{N-1}\ldots \nu_{1} \end{array}$$ (9)

This last equation shows a very general result: the overall *n*_*H*_ of a cascade is a multiplicative combination of the *ν*_*i*_ of each module. Therefore, the effective response coefficients allow us to characterize the relative contribution of each layer to the overall system’s ultrasensitivity.

It is worth noting that the factor two in Eq 9 arises from the average response coefficient of a reference hyperbolic curve that appears in the original definition of the Hill coefficient (see Eq 6). Hence, the ultrasensitivity character of the cascade remains a system level feature, as it involves the product of the effective coefficient of all layers, in units of the logarithmic amplification coefficient of a single reference hyperbolic curve.

### The effect of the Hill’s working range in multi-tiered systems

According to Eq (9) the HWR of a module delimits the relevant region of inputs over which local-ultrasensitivity features of module’s response functions are combined to build up the overall system behavior. It is thus a significant parameter to get insight into the overall ultrasensitivity of multilayered structures. In this section we show, for different types of dose-response curves, how the HWR depends on the way that cascade layers are actually coupled.

#### Composition of Hill functions

Let’s start by considering two coupled ultrasensitive modules of the Hill type. As explained above, two different regimes can be identified depending on whether the upstream module’s maximum output is large enough to fully activate the downstream unit or not (Fig 2A or 2B, respectively).

#### Downstream saturation regime

When *O*_1,*max*_ ≫ *EC*50_2_ (Fig 2A), *X*10_2_ and *X*90_2_ are equal to the respective *EC*10_2_ and *EC*90_2_ levels. This corresponds to what we call *The downstream saturation regime*. In this scenario, the HWR of module-2 does not differ from the one corresponding to the isolated case, and thus $\nu_{2}={\langle R_{2}\rangle }_{X10_{2},X90_{2}}=n_{2}^{is}/2$. In the last expression $n_{2}^{is}$ refers to the Hill coefficient of module-2 when considered in isolation. On the other hand the HWR of module-1 tends to be located at low input-values for increasing levels of the ratio *O*_1,*max*_/*EC*50_2_. In this region, the response coefficient of the Hill functions reaches its highest values, $R_{1}\approx n_{1}^{is}$ (see Fig 1C). Thus, when calculating the response coefficient, we obtained $\nu_{1}={\langle R_{1}\rangle }_{X10_{1},X90_{1}}=n_{1}^{is}$. Finally, from Eq 9, it follows that

$$n_{H}=2.\nu_{1}.\nu_{2}=2.n_{1}^{is}.n_{2}^{is}/2=n_{1}^{is}.n_{2}^{is}.$$

Therefore, the cascade behaves multiplicatively in this regime, which is consistent with Ferrell’s results [13]

#### Upstream saturation regime

When *O*_1,*max*_ ≲ *EC*50_2_ (ie, when the upstream module’s maximal output does not fully activate the downstream module), different behaviors could arise depending on module-2 ultrasensitivity characteristics at low input values.

**Fig 3:**
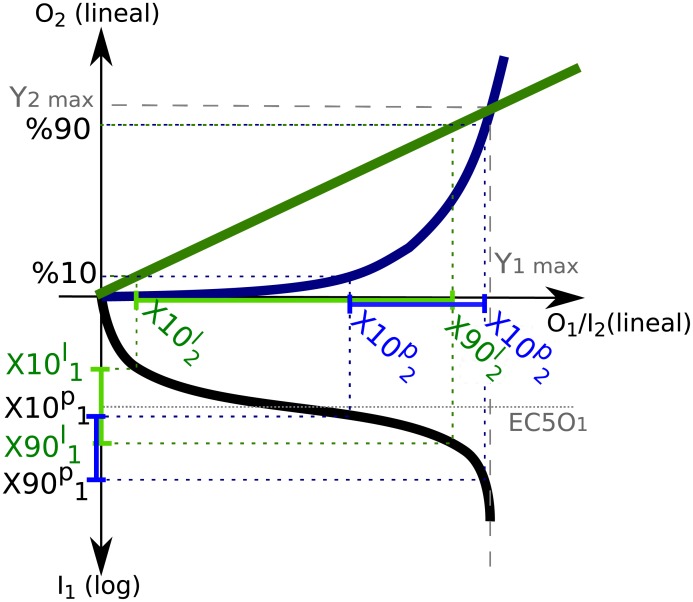
Schematic diagrams of the response function when composing a Hill function in module-1, with a linear function (in green) or a power function (in blue) in module-2.

For instance, let’s consider that the dose-response of module-2 has an *n*_2_ = 1. This means that the response is linear at low input values (see Fig 3). This linearity causes $X10_{2}^{l}$ and $X90_{2}^{l}$ (*X*10_2_ and *X*90_2_ of the linear curve) to match the %10 and %90 of *O*_1,*max*_. Therefore, $X10_{1}^{l}$ and $X90_{1}^{l}$ equal *EC*10_1_ and *EC*90_1_, respectively, centering the HWR around the *EC*50_1_. Furthermore, as a result of the linearity displayed by the response function of module-2 in this regime, the system’s overall behavior relies exclusively on module-1’s ultrasensitivity and, given the linearity of module-2, it shows a multiplicative behavior. Applying Eq 9

$$\begin{array}{c} n_{H}=2\overset{\nu_{2}}{\overset{︷}{{\left\langle R_{2}\right\rangle }_{X10_{2},X90_{2}}}}\overset{\nu_{1}}{\overset{︷}{{\left\langle R_{1}\right\rangle }_{X10_{1},X90_{1}}}}=2\overset{\nu_{2}}{\overset{︷}{1}}\overset{\nu_{1}}{\overset{︷}{n_{1}/2}}=n_{1} \end{array}$$

On the other hand, when *n*_2_ > 1, the response of module-2 follows a power-law at low input values (see Fig 3). This non-linearity produces a shift in the working range of module-2 towards higher values, which centers the HWR of module-1 [*X*10_1_, *X*90_1_] around input values higher than *EC*50_1_. Furthermore, given that *R*_1_ decreases with *I*_1_ (see Fig 1C), the shift in module-1’s working range results in *ν*_1_ = 〈*R*_1_〉_*X*10_1_, *X*90_1__ < *n*_1_/2, and consequently,

$$\begin{array}{c} n_{H}=2\overset{\nu_{2}}{\overset{︷}{{\left\langle R_{2}\right\rangle }_{X10_{2},X90_{2}}}}\overset{\nu_{1}}{\overset{︷}{{\left\langle R_{1}\right\rangle }_{X10_{1},X90_{1}}}}<2\overset{\nu_{2}}{\overset{︷}{n_{2}}}\overset{\nu_{1}}{\overset{︷}{n_{1}/2}}=n_{2}n_{1} \end{array}$$

Therefore, whenever *n*_2_ > 1, for the upstream saturation condition (*O*_1,*max*_ < *EC*50_2_), we found that the system’s ultrasensitivity is submultiplicative, consistent with Ferrell’s results [13].

#### Golbeter-Koshland functions composition

The exact functional form of the response curve of an ultrasensitive module could affect the overall system’s ultrasensitivity in cascade architectures. In particular, we found that a system composed of two modules characterized by Golbeter-Koshland, GK, rather than Hill response functions [9] shows a qualitatively different behavior.

**Fig 4:**
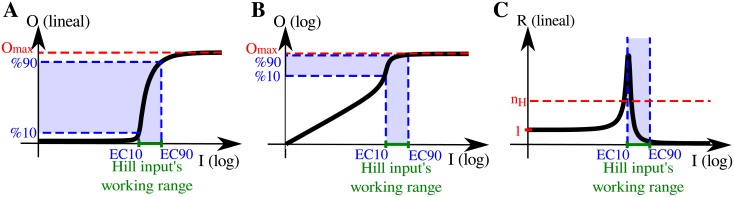
Schematic representations of Goldbeter-Koshland dose-response curves with *K*_1_ ≳ 1 and *K*_2_ ≪ 1 (see equation in S1 Text) shown in log-linear scale (A) and in log-log scale (B). The corresponding response coefficient (C) shows no local ultrasensitivity for low input values (i.e. *R* ∼ 1), but displays high local ultrasensitivity, even larger than the module’s Hill coefficient *n*_*H*_, for intermediate input regions.

GK functions appear in the mathematical characterization of enzymatic covalent modification cycles (such as phosphorylation-dephosphorylation) when the enzymes are saturated (see S1 Text). The detailed functional form of the transfer function depends on the operating regimes of the phosphorylation and dephosphorylation processes [29]. For cases where the phosphatases, but not the kinases, work in saturation, we observed that GK functions present input regions with response coefficients higher than their overall *n*_*H*_ [21] (see Fig 4A–4C). Hence, whenever their HWR is located in the region of largest local ultrasensitivity, these functions are able to contribute with more effective ultrasensitivity than what is expected from their global ultrasensitivity descriptors. As a result, cascades involving GK functions may exhibit supra-multiplicative behavior.

**Fig 5:**
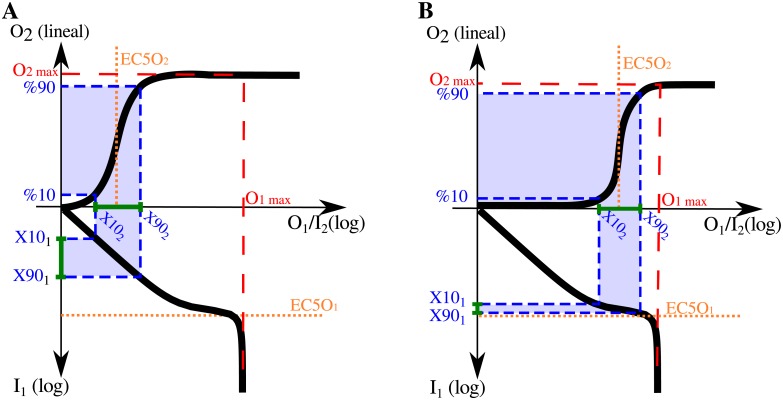
Schematic response function diagrams for two different compositions of two GK ultrasensitive modules are shown in panels (A) and (B). Axes were arranged as explained in Fig 2’s caption. In panel (A) *O*_1,*max*_ ≫ *EC*50_2_, and module-1’s HWR covers the input region below *EC*50_1_, a region in which the curve shows no local ultrasensitivity (*R*_1_ = 1). In panel (B) we show a special scenario where the *O*_2,*max*_/*EC*50_2_ ratio was tuned in order to set module-1’s HWR in its most ultrasensitive region.

For a two tier arrangement of this kind of modules, in downstream saturation regime, module-1’s HWR is set in its linear response regime (i.e. *R*_1_ = 1), and thus the GK function does not contribute to the overall system’s ultrasensitivity (Fig 5A). However, we found that there is a particular *O*_1,*max*_/*EC*50_2_ ratio value for which module-1’s HWR spans the most ultrasensitive region of the module’s transfer function, producing an effective response coefficient, *ν*_2_, that is larger even than the overall ultrasensitivity of the second GK curve in isolation (i.e. *ν*_2_ ≥ *n*_2_). In this case, the system exhibits a supra-multiplicative behavior: $n_{H}=2.\nu_{1}.\nu_{2}>n_{1}^{is}.n_{2}^{is}$ (Fig 5B).

Comparing the Hill and GK cases, our analysis highlights the impact of the detailed functional form of a module’s response curve on the overall system’s ultrasensitivity. Thus, local ultrasensitivity features of the involved transfer functions are of utmost importance in cascades.

### Disentangling the contribution to the observed ultrasensitivity of HWR and sequestration effects

As we have shown in the preceding sections, the shift of HWRs as a consequence of module coupling could be at the core of the system’s ultrasensitivity. In addition, sequestration effects, affecting free active enzyme concentrations due to the formation of intermediary complexes, could also play an important role in this respect [22–24]. Sequestration and dynamic range constraints not only contribute with their individual complexity, but also usually occur together, thus making it more difficult to identify their individual effective contribution to the system’s overall ultrasensitivity.

In order to determine the impact of these two factors, we simultaneously considered two approximations of the system under study (see S1 Fig). For a given model, we first considered the mathematical composition of each module’s response function (e.g. for a MAPK cascade $F_{MAPK}^{non-seq}\left(x\right)=f_{MAPK}^{is}(f_{MAPKK}^{is}(f_{MAPKKK}^{is}\left(x\right)))$, see S1B Fig). We called this expression *F*^*non*—*seq*^, since sequestration effects were completely neglected in this transfer function. In addition, we also estimated the response function *F*^*seq*^, obtained by numerical integration of the the corresponding mechanistic model of the cascade S1C Fig). *F*^*seq*^ includes, if present, sequestration effects.

In this way, the first estimation, *F*^*non*—*seq*^, allowed us to analyze to what extent the existence of HWRs impinges on ultrasensitivity features of the cascade arrangement. *F*^*seq*^ not only incorporates HWR resetting effects, but also helps assessing the impact of potential sequestration effects in the system (see S1 Fig).

### Ultrasensitivity in O’Shaughnessy *et al*. models

**Fig 6:**
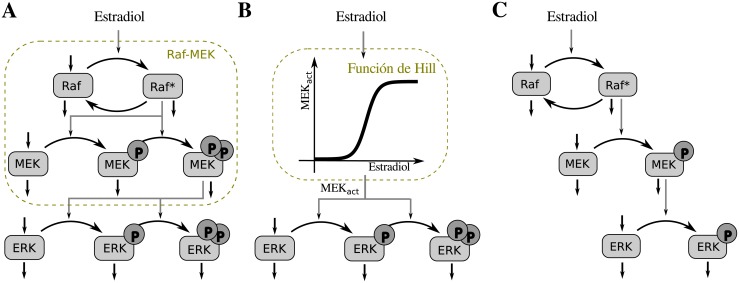
O’Shaughnessy et al. cascades scheme. The three models of the mammalian MAPK cascade expressed in yeast. Represented with a dual-step phosphorylation (A), with the Raf and MEK layers replaced by a Hill Function (B) whose parameters were obtained by fitting the function to the active MEK dose-response, and a MAPK cascade with a single-step phosphorylation (C). In each case, estradiol is the input and dually phosphorylated ERK is the output.

In this section, we revisited three different mathematical models proposed by O’Shaughnessy *et al*. (Fig 6) to disentangle the origin of the ultrasensitive behavior they observed in a mammalian MAPK cascade expressed in yeast [28]. In particular, we show how we used the methodology and concepts introduced so far to better understand mathematical descriptions of real cascades.

The three analyzed mathematical models were a three-tier dual-step phosphorylation cascade, a phenomenological scheme that lumps together the Raf and MEK layers, and finally a three-layer single-step phosphorylation cascade (Fig 6(A), 6(B) and 6(C), respectively, for model details see S2 Text).

#### Ultrasensitivity in the dual-step phosphorylation model

A sketch of this model is shown in Fig 6A. We defined the output of a module and the input to the next as the total active form of a species, including complexes with the next layer’s substrates. However, we excluded complexes formed by same-layer components (such as a complex between the phosphorylated kinase and its phosphatase), since these species are ‘internal’ to each module. By doing this, we were able to consistently identify layers with modules (the same input/output definition was used by Ventura *et al* [25]).

The analysis of *F*^*non*—*seq*^, i.e. the mathematical composition of the response functions of the isolated modules, allowed us to assess the effects of coupling modules (the *cascading effect*). We observed that module coupling resulted in HWRs so that the overall system ultrasensitivity was $n_{H}^{non-seq}=3.91$. This value was lower than the product of each module’s Hill coefficient ($n_{1}^{is}n_{2}^{is}n_{3}^{is}=5.02$), and thus the cascade behaved sub-multiplicatively.

**Fig 7:**
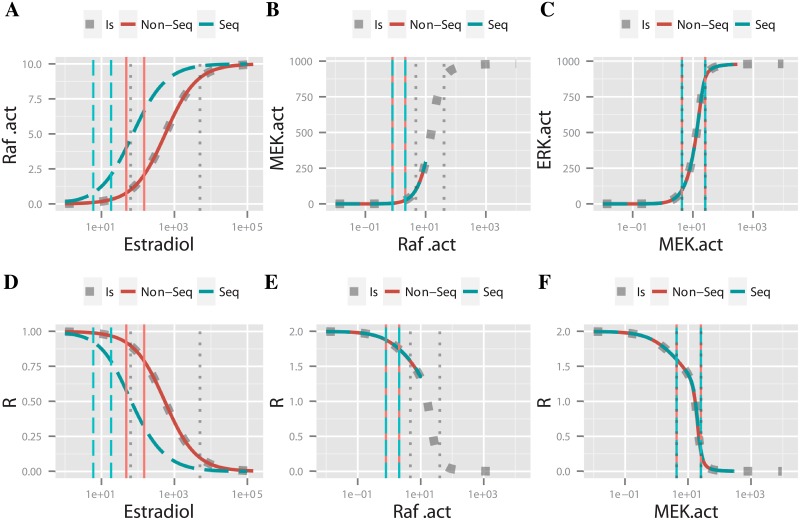
Dose-response analysis for the dual step phosphorylation model. Transfer functions for each of the three layers of the MAPK cascade (A-C), obtained considering for each layer i) the isolated module (Is, dotted blue), ii) a mechanistic implementation of the model (Seq, dashed-turquoise) and iii) the mathematical composition of isolated response functions (Non-Seq, continous red). The corresponding response coefficient curves are shown in panels (D-F). Turquoise dashed vertical lines show the *X*10_*i*_ and *X*90_*i*_ values of each layer (i.e. mechanistic scheme), while red solid vertical lines mark the layer’s *X*10_*i*_ and *X*90_*i*_ associated to the composition of response curves of each module (i.e. *F*^*non*—*seq*^).

Notably, sequestration did not affect the ultrasensitivity of this system: for both implementations of the system, *F*^*non*—*seq*^ and *F*^*seq*^, we obtained $n_{H}^{Seq}=n_{H}^{Non-Seq}=3.91$. To understand why this is the case, we refer to Fig 7, which shows the estradiol-act:Raf, act:Raf-act:MEK, and act:MEK-act:ERK response functions for the dual-step phosphorylation model. The effect of sequestration was negligible for the MAPKK and MAPK layers, given the overlap observed for the corresponding *F*^*non*—*seq*^ and *F*^*seq*^ response functions (Fig 7B and 7C). Only for the MAPKKK layer, sequestration produced a shift between these curves (Fig 7A). However, this shift did not result in changes in the ultrasensitivity of the system because, unexpectedly, the corresponding HWRs changed in a manner that compensated the sequestration effect, so that the effective ultrasensitive coefficients remained unchanged (i.e. $\nu_{Estradiol-Raf.act}^{Seq}=\nu_{Estradiol-Raf.act}^{Non-Seq}$).

Hence, we conclude that in this particular mathematical model, even though sequestration effects existed, the overall sub-multiplicative behavior was only due to a shift in the position of the HWR for the first and second layers of the cascade.

We obtained similar conclusions from our analysis of the single step phosphorylation cascade (data not shown).

#### The Raf-MEK lumped model

In order to support the hypothesis that a cascading effect contributed to the system ultrasensitivity, O’Shaughnessy *et* al. [28] analyzed the MAPK cascade with the Raf-MEK levels replaced by one Hill function (Fig 6 panels A-B). They observed that such a reduced two-layer cascade had a lower ultrasensitivity than the original with three layers, and proposed that the presence of intermediate species (MEKpp complexes in the second layer, omitted after the replacement) were the origin of this ultrasensitivity.

In order to understand the ultimate origin of this behavior, we compared the local and global ultrasensitivity descriptors in both cascades. Like O’Shaughnessy *et al*. [28], we observed a reduction of the cascade’s ultrasensitivity when the Raf and MEK levels were aggregated into a Hill function, from *n*_*H*_ = 3.91 to $n_{H}^{fhill}=2.7$. As we explained above (Eq 9), Hill coefficients can be written as a function of the effective response coefficients. For the original and the reduced cascades:

$$\begin{array}{ccc} n_{H} & = & 2\;\nu_{Raf}\;\nu_{MEK}\;\nu_{ERK}=2\;\nu_{Raf-MEK}\;\nu_{ERK} \end{array}$$ (10)

$$\begin{array}{ccc} n_{H}^{fhill} & = & 2\;\nu_{Raf-MEK}^{fhill}\;\nu_{ERK}^{fhill} \end{array}$$ (11)

Given that the Hill function used to lump the two top layers fits rather well the Estradiol-MEK curve (Fig 6A), the HWR of the ERK layer was the same in the lumped and original three-layer cascade models. Therefore $\nu_{ERK}=\nu_{ERK}^{fhill}$. Then, the fact that $n_{H}>n_{H}^{fhill}$ meant necessarily that $\nu_{Raf-MEK}>\nu_{Raf-MEK}^{fhill}$.

Hence, the observed reduction in the overall ultrasensitivity of the reduced-layer model was due to a reduction of the effective response coefficient of the Hill approximating function used to aggregate the Raf-MEK layers, $\nu_{Raf-MEK}^{fhill}$, relative to the effective response coefficient of the originial Estradiol-MEK response curve, *ν*_*Raf*—*MEK*_ (Fig 6B). We calculated the effective response coefficient in each case, obtaining a *ν*_*Raf*—*MEK*_ = 1.58 and a *ν*_*Hill func*_ = 1.09, consistent with our expectations. The combined Raf-MEK layers have a Hill coefficient of $n_{H}^{Raf-MEK}=n_{H}^{fHill}=1.14$. This indicated that while the Raf-MEK system contributes to the original cascade with an ultrasensitivity higher than its Hill coefficient, this was not the case for the reduced-layer model.

**Fig 8:**
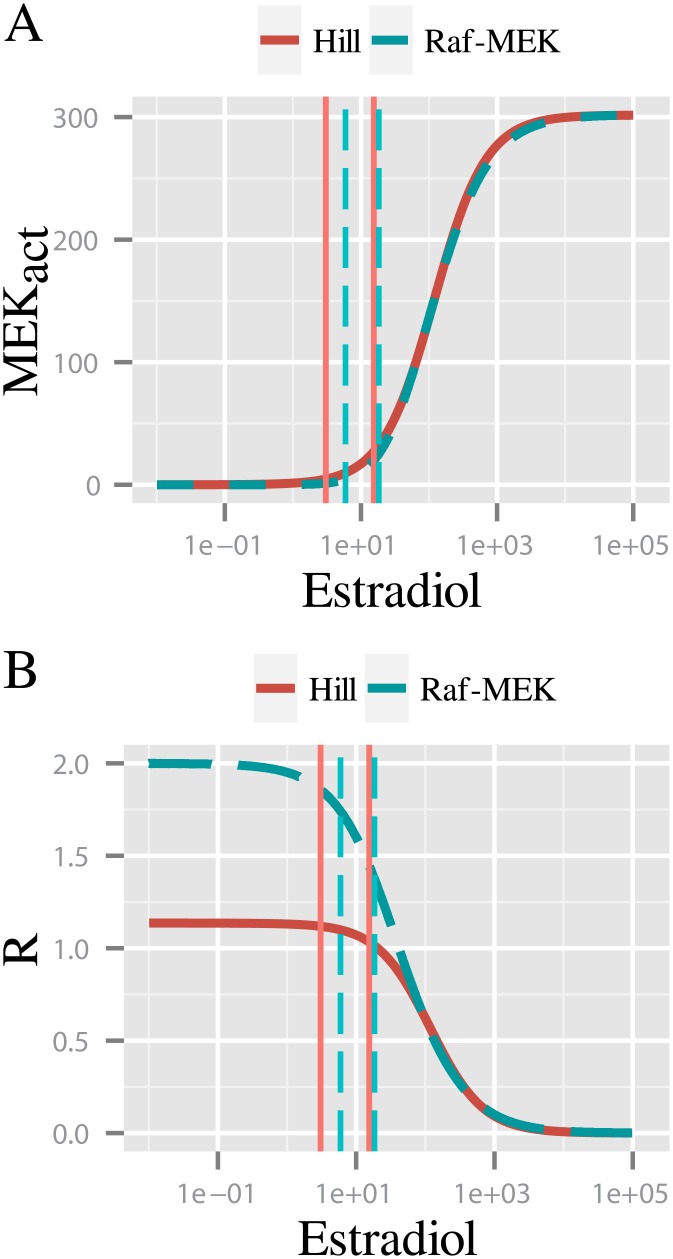
Fitting by a Hill function may obscure relevant behaviors. Dose-response curve of active MEK in O’Shaughnessy model compared with its fit by a Hill function (A). Respective response coefficient (B). Even though the dose-responses of active MEK and the Hill function appear to be similar (A), there are strong differences in their local ultrasensitivity.

The cause of this behavior becomes clear analyzing Fig 8. Even though the dose-response of active MEK and the Hill approximating function appeared to be identical, there were strong differences in their local ultrasensitivity behavior. This was particularly true in the low input region, where the HWR happened to be located. In this region, the active MEK curve presented a local ultrasensitivity larger than the Hill function counterpart. Therefore, the replacement by a Hill function produced a reduction in the corresponding Hill coefficient. In this way, despite the high-quality of the fitting adjustment (Residual Standard Error = 2.6), the Hill function approximation introduced significant alterations in the system’s ultrasensitivity. This may be considered a technical defect.

This is a remarkable result as it means that a well approximating function from the standard minimization procedure perspective, might have a non-trivial impact on the qualitative conclusions to be drawn from a system’s behavior.

#### The single-step phosphorylation model

In order to probe the origin of the ultrasensitivity observed in the original cascade (Fig 6A), O’Shaughnessy et al. constructed an auxiliary model in which dual-step phosphorylation layers where replaced by single-step phosphorylation layers. This simplified cascade was still ultrasensitive. Because in this new setting the cascade lacked multiple activation processes, competitive inhibition, and zero-order ultrasensitivity (due to the absence of phosphatases), they claimed that there were no ultrasensitivity sources other than the kinase-cascade architecture itself. Thus, they proposed that a *cascading effect* generated ultrasensitivity *de-novo*.

**Fig 9:**
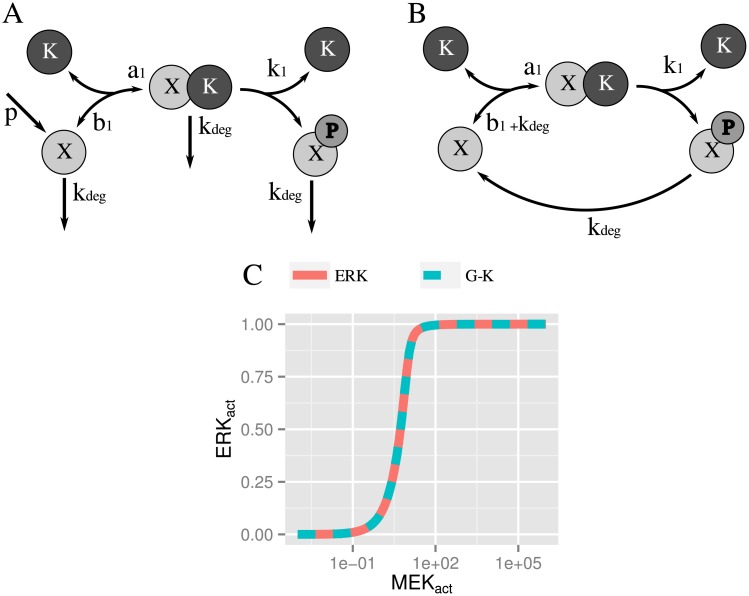
Equivalence between a single-step layer in O’Shaughnessy model and a covalent modification cycle. O’Shaughnessy et al. single-step layer (A) and the equivalent covalent modification cycle (B). (C) Steady state transfer functions of ERK layer in isolation of the O’Shaughnessy single-step cascade (blue dashed line), compared to a centered Goldbeter-Function with equivalent parameters (red solid line) (*K*_1_ = 0.04 and *K*_2_ = 1000, see S1 Text).

When we re-analyzed this simplified model, despite their claim, we observed that the MEK and ERK modules considered in isolation were ultrasensitive (*n*^*MEK*^ = 1.54 and *n*^*ERK*^ = 1.76). Synthesis and degradation were the key factors to understand the origin of their ultrasensitivity. We realized that these layers (Fig 9A) were in fact mathematically analogous to a covalent cycle (Fig 9B) because there was an implicit channel from the activated protein towards its inactive form via the degradation of the active protein and the production of the inactive form. Given that degradation is a linear reaction with respect to the amount of activated protein, its mathematical description is equivalent to a dephosphorylation reaction operating in a first order regime. Equivalently, it can be considered as a limit case where the complex formed by the active protein and phosphatase instantly disassembles (i.e. *K*_2_ → ∞)

Therefore, the one-step system depicted in Fig 9 could in fact be described by a Goldbeter-Koshland (G-K) [9] function with

$$\begin{array}{c} K_{1}=\frac{K_{deg}+b_{1}+k_{1}}{X_{T}a_{1}}\;\;\;and\;\;\;K_{2}\gg 1 \end{array}$$ (12)

We plotted in Fig 9C the steady state transfer function of the ERK module in isolation and the corresponding centered G-K function (see S1 Text). There was a clear agreement between both functions. In the light of these results, we concluded that the single-step cascade’s ultrasensitivity did not arise *de novo* from a *cascading effect* but from a *hidden* G-K ultrasensitivity process in the MEK and ERK layers.

## Discussion

The study of signal transmission and information processing inside the cell has been, and still is, an active field of research. In particular, the analysis of signaling cascades has received a lot of attention as they are well-conserved motifs that can be found in many cell fate decision systems. The aim of this paper was to propose a framework to characterize and better understand mathematical models used to study real biological systems. For a given mathematical model, the methodology we described, allowed us to disentangle the origin of the predicted ultrasensitivity behavior in terms of HWR repositioning and/or sequestration effects acting on the modular cascade architecture of interest. In this respect, even though we have not addressed the general and important problem of resolving the working principles acting on a given real cascade, we did provide a useful tool for modelers to better understand and perform educated choices between modeling alternatives.

It is also worth noting that dynamical features of signal transduction systems might play an important role on the system-level displayed behavior. In order to analyze signaling cascades whenever this happens, one should not only deal with the coupling of modular input-output response functions but also with their characteristic time-scales. Despite of this, a steady state analysis, such as the one presented here, still offers useful information and remains a sensible approximation whenever there is no effective time-scale separation between modules.

In this work we have found a mathematical expression (Eq 6) that linked local and global ultrasensitivity descriptors in a fairly simple way. Moreover, we have provided a general result to handle the case of a linear arrangement of an arbitrary number of such modules (Eq 9). The value of the resulting expression lies in that not only it captured previous results, like Ferrell’s inequality, but also threw light on the mechanisms involved in ultrasensitivity generation. For instance, the existence of supramultiplicative behaviors in signaling cascades have been reported by several authors [23, 28] but in many cases the ultimate origin of supramultiplicativity remained elusive. Our framework suggested in a simple way a general scenario where supramultiplicative behavior arises. This could occur when, for a given module, the corresponding HWR is located in an input region with a local ultrasensitivity higher than the global ultrasensitivity of the respective dose-response curve.

Notably, within the proposed analytical framework, we were able to decompose the overall global ultrasensitivity in terms of a product of single layer effective response coefficients. These new parameters were calculated as local-sensitivity values averaged over meaningful working ranges, the HWRs, which permitted the assessment of the effective contribution of each module to the system’s overall ultrasensitivity. Of course, the reason why we could present an exact general equation for a system-level feature in terms of individual modular information was that in fact system-level information was implicitly used in the definition of the HWR that entered Eq 9. The specific coupling between ultrasensitive curves sets the corresponding HWRs, thus determining the effective contribution of each module to the cascade’s ultrasensitivity. This process, which we called *HWR setting*, has already been noticed by several authors [13, 20, 21, 23, 30–32], but this is the first time that a mathematical framework, like the one we present here, has been proposed for it.

We used our methodology to revisit the different mathematical models considered by O’Shaughnessy et al. to analyze their tunable synthetic MAPK system [28], and we were able to bring a new perspective to the conclusions that could be drawn from such mathematical constructs. For instance, we proved that sequestration effects played no role in the observed system ultrasensitivity for the dual-step and single-step phosphorylation models. We were also able to analyze the auxiliary model in which the Raf and MEK layers were replaced by a Hill function that was coupled to the ERK layer. In this case, even though the original Estradiol-MEK input-output response curve could be relatively well fitted and global ultrasensitivity features were well captured, the mere replacement by a Hill function produced a strong decrease in the system’s ultrasensitivity. We found that the functional form of the Hill function failed to reproduce the original local ultrasensitivity features that were in fact the ones that, due to the particular HWR acting in this case, were responsible for the overall systems ultrasensitivity behavior. The analyzed case was particularly relevant, since it highlighted potential technical problems that could arise as a consequence of the inclusion of approximating functions in mathematical models.

## Conclusions

In this article we provided a framework for characterizing mathematical models used to describe real biological systems of ultrasensitive character. We presented a mathematical link between global and local ultrasensitivity estimators for a sigmoidal unit and generalized these results for a cascade of such units. Using the introduced concept of HWR, the overall system’s ultrasensitivity could be defined in terms of effective contributions of each cascade layer. Moreover, we were able to explain the origin of the ultrasensitivity in a given mathematical model in terms of HWR repositioning and/or sequestration effects.

Our framework may help to understand the origin of ultrasensitivity in general multilayer structures, and in this sense it could be useful in the design of synthetic systems [33–35]. For instance, given that a specific HWR setting (targeting the region of maximal local ultrasensitivity of a given unit in a cascade) is a key factor in producing high overall ultrasensitivity, our methodology can be used to guide the tuning of a single module’s features, as well as its coupling with other units to form a cascade.

## Supporting information

S1 Text (PDF)

S2 Text (PDF)

S1 FigModular and system representation of a MAP kinase cascade.Each layer in isolation is composed of single or multiple covalent cycles. Each dose-response curve can be ultrasensitive as a result of zero-order mechanisms and/or multi-activation processes (A). The cascade transfer function, in a scenario in which sequestration is not taken into account (*F*^*non*—*seq*^), may be obtain by the mathematical composition of each module’s transfer function considered in isolation $f_{i}^{is}$(B). When the sequestration effect is taken into account, the layers embedded in the MAP kinase cascade may have a different dose-response curve from the isolated case (C).(EPS)

## References

[1] Ferrell JE, Ha SH. Ultrasensitivity part III: cascades, bistable switches, and oscillators. Trends in biochemical sciences. 2014;39(12):612–618. 10.1016/j.tibs.2014.10.002 25456048 PMC4254632

[2] Zhang Q, Bhattacharya S, Andersen ME. Ultrasensitive response motifs: basic amplifiers in molecular signalling networks. Open biology. 2013;3(4):130031 10.1098/rsob.130031 23615029 PMC3718334

[3] Angeli D, Ferrell JE, Sontag ED. Detection of multistability, bifurcations, and hysteresis in a large class of biological positive-feedback systems. Proceedings of the National Academy of Sciences. 2004;101(7):1822–1827. 10.1073/pnas.0308265100 PMC357011 14766974

[4] Ferrell JE Jr, Xiong W. Bistability in cell signaling: How to make continuous processes discontinuous, and reversible processes irreversible. Chaos: An Interdisciplinary Journal of Nonlinear Science. 2001;11(1):227–236. 10.1063/1.1349894 12779456

[5] Srividhya J, Li Y, Pomerening JR. Open cascades as simple solutions to providing ultrasensitivity and adaptation in cellular signaling. Physical biology. 2011;8(4):046005 10.1088/1478-3975/8/4/046005 21566270 PMC3151678

[6] Kholodenko BN. Negative feedback and ultrasensitivity can bring about oscillations in the mitogen-activated protein kinase cascades. European Journal of Biochemistry. 2000;267(6):1583–1588. 10.1046/j.1432-1327.2000.01197.x 10712587

[7] Buchler NE, Louis M. Molecular titration and ultrasensitivity in regulatory networks. Journal of molecular biology. 2008;384(5):1106–1119. 10.1016/j.jmb.2008.09.079 18938177

[8] Buchler NE, Cross FR. Protein sequestration generates a flexible ultrasensitive response in a genetic network. Molecular systems biology. 2009;5(1):272 10.1038/msb.2009.30 19455136 PMC2694680

[9] Goldbeter A, Koshland DE. An amplified sensitivity arising from covalent modification in biological systems. Proceedings of the National Academy of Sciences. 1981;78(11):6840–6844. 10.1073/pnas.78.11.6840 PMC349147 6947258

[10] Ferrell JE, Ha SH. Ultrasensitivity part I: Michaelian responses and zero-order ultrasensitivity. Trends in biochemical sciences. 2014;39(10):496–503. 10.1016/j.tibs.2014.08.003 25240485 PMC4214216

[11] Ferrell JE, Ha SH, et al Ultrasensitivity part II: multisite phosphorylation, stoichiometric inhibitors, and positive feedback. Trends in biochemical sciences. 2014;39(11):556–569. 10.1016/j.tibs.2014.09.003 25440716 PMC4435807

[12] Ferrell JE. Tripping the switch fantastic: how a protein kinase cascade can convert graded inputs into switch-like outputs. Trends in biochemical sciences. 1996;21(12):460–466. 10.1016/S0968-0004(96)20026-X 9009826

[13] Ferrell JE. How responses get more switch-like as you move down a protein kinase cascade. Trends in biochemical sciences. 1997;22(8):288–289. 10.1016/S0968-0004(97)82217-7 9270299

[14] Markevich NI, Hoek JB, Kholodenko BN. Signaling switches and bistability arising from multisite phosphorylation in protein kinase cascades. The Journal of cell biology. 2004;164(3):353–359. 10.1083/jcb.200308060 14744999 PMC2172246

[15] Gunawardena J. Multisite protein phosphorylation makes a good threshold but can be a poor switch. Proceedings of the National Academy of Sciences of the United States of America. 2005;102(41):14617–14622. 10.1073/pnas.0507322102 16195377 PMC1253599

[16] Rippe K. Analysis of protein-DNA binding at equilibrium. BIF futura. 1997;12:20–26.

[17] Kholodenko BN, Hoek JB, Westerhoff HV, Brown GC. Quantification of information transfer via cellular signal transduction pathways. FEBS letters. 1997;414(2):430–434. 10.1016/S0014-5793(97)01018-1 9315734

[18] Keshet Y, Seger R. The MAP kinase signaling cascades: a system of hundreds of components regulates a diverse array of physiological functions. MAP Kinase Signaling Protocols: Second Edition. 2010;p. 3–38. 10.1007/978-1-60761-795-2_1 20811974

[19] Huang CY, Ferrell JE. Ultrasensitivity in the mitogen-activated protein kinase cascade. Proceedings of the National Academy of Sciences. 1996;93(19):10078–10083. 10.1073/pnas.93.19.10078 PMC38339 8816754

[20] Brown GC, Hoek JB, Kholodenko BN. Why do protein kinase cascades have more than one level? Trends in biochemical sciences. 1997;22(8):288 10.1016/S0968-0004(97)82216-5 9270298

[21] Altszyler E, Ventura A, Colman-Lerner A, Chernomoretz A. Impact of upstream and downstream constraints on a signaling module’s ultrasensitivity. Physical biology. 2014;11(6):066003 10.1088/1478-3975/11/6/066003 25313165 PMC4233326

[22] Blüthgen N, Bruggeman FJ, Legewie S, Herzel H, Westerhoff HV, Kholodenko BN. Effects of sequestration on signal transduction cascades. Febs Journal. 2006;273(5):895–906. 10.1111/j.1742-4658.2006.05105.x 16478465

[23] Rácz É, Slepchenko BM. On sensitivity amplification in intracellular signaling cascades. Physical biology. 2008;5(3):036004 10.1088/1478-3975/5/3/036004 18663279 PMC2675913

[24] Wang G, Zhang M. Tunable ultrasensitivity: functional decoupling and biological insights. Scientific reports. 2016;6. 10.1038/srep20345 PMC4742884 26847155

[25] Ventura AC, Sepulchre JA, Merajver SD. A hidden feedback in signaling cascades is revealed. PLoS Comput Biol. 2008;4(3):e1000041 10.1371/journal.pcbi.1000041 18369431 PMC2265423

[26] Del Vecchio D, Ninfa AJ, Sontag ED. Modular cell biology: retroactivity and insulation. Molecular systems biology. 2008;4(1):161 10.1038/msb4100204 18277378 PMC2267736

[27] Ventura AC, Jiang P, Van Wassenhove L, Del Vecchio D, Merajver SD, Ninfa AJ. Signaling properties of a covalent modification cycle are altered by a downstream target. Proceedings of the National Academy of Sciences. 2010;107(22):10032–10037. 10.1073/pnas.0913815107 PMC2890436 20479260

[28] O’Shaughnessy EC, Palani S, Collins JJ, Sarkar CA. Tunable signal processing in synthetic MAP kinase cascades. Cell. 2011;144(1):119–131. 10.1016/j.cell.2010.12.014 21215374 PMC3035479

[29] Gomez-Uribe C, Verghese GC, Mirny LA. Operating regimes of signaling cycles: statics, dynamics, and noise filtering. PLoS Comput Biol. 2007;3(12):e246 10.1371/journal.pcbi.0030246 18159939 PMC2230677

[30] Blüthgen N, Herzel H. Map-kinase-cascade: switch, amplifier or feedback controller. In: 2nd Workshop on Computation of Biochemical Pathways and Genetic Networks; 2001. p. 55–62.

[31] Blüthgen N, Herzel H. How robust are switches in intracellular signaling cascades? Journal of theoretical biology. 2003;225(3):293–300. 10.1016/S0022-5193(03)00247-9 14604583

[32] Tsvetanova NG, Trester-Zedlitz M, Newton BW, Riordan DP, Sundaram AB, Johnson JR, et al GPCR endocytosis confers uniformity in responses to chemically distinct ligands. Molecular Pharmacology. 2016;p. mol–116. 10.1124/mol.116.106369 PMC5267521 27879340

[33] Zechner C, Seelig G, Rullan M, Khammash M. Molecular circuits for dynamic noise filtering. Proceedings of the National Academy of Sciences. 2016;113(17):4729–4734. 10.1073/pnas.1517109113 PMC4855548 27078094

[34] Kittleson JT, Wu GC, Anderson JC. Successes and failures in modular genetic engineering. Current opinion in chemical biology. 2012;16(3):329–336. 10.1016/j.cbpa.2012.06.009 22818777

[35] Ang J, Harris E, Hussey BJ, Kil R, McMillen DR. Tuning response curves for synthetic biology. ACS synthetic biology. 2013;2(10):547–567. 10.1021/sb4000564 23905721 PMC3805330

