# Supplementary material for: Ultrasensitivity in signaling cascades revisited: Linking local and global ultrasensitivity estimations
Source: PLoS One. 2017 Jun 29;12(6):e0180083. doi: 10.1371/journal.pone.0180083 (PMC5491127; doi:10.1371/journal.pone.0180083)
Supplement: S1 Text — (PDF) [file pone.0180083.s001.pdf]

Supplementary Information Text S1

Ultrasensitivity on signaling cascades revisited: Linking local and global ultrasensitivity estimations

Edgar Altszyler[1], Alejandra C. Ventura[2], Alejandro Colman-Lerner[2] and Ariel Chernomoretz *[3,4]

[1]Departamento de Computación, Universidad de Buenos Aires - CONICET.
[2]Departamento de Fisiología, Biología Molecular y Celular, Facultad de Ciencias Exactas y Naturales, Universidad de Buenos Aires and Instituto de Fisiología, Biología Molecular y Neurociencias (IFIBYNE-CONICET), Ciudad Universitaria Pabellón II, C1428EHA Buenos Aires, Argentina.
[3]Departamento de Física FCEN UBA - IFIBA CONICET.
[4]Fundación Instituto Leloir.

Goldbeter-Koshland function

The Goldbeter-Koshland function [?] is used to describe the steady state concentration of a protein affected by phosphorylation/de-phosphorylation modifications (schematized in Fig 1).

It's transfer function is given by the expression:

$$G(x, K_1, K_2) = \frac{(x-1) - K_2\Big(x + \frac{K_1}{K_2}\Big) + \Big(x - 1 - K_2\Big(x + \frac{K_1}{K_2}\Big) - 4K_2(x-1)x\Big)^{1/2}}{2(x-1)} \tag{1}$$

with

$$x = \frac{k_1 K_T}{k_2 P_T} \;\; , \;\; K_1 = \frac{b_1 + k_1}{X_T a_1} \;\; and \;\; K_2 = \frac{b_2 + k_2}{X_T a_2} \tag{2}$$

where X_T, K_T and P_T are the total concentration of proteins X, K and P, respectively. K_1 and K_2 are the phosphorylation and de-phosphorylation Michaelis constants divided by X_T, respectively.

*Corresponding author: ariel@df.uba.ar

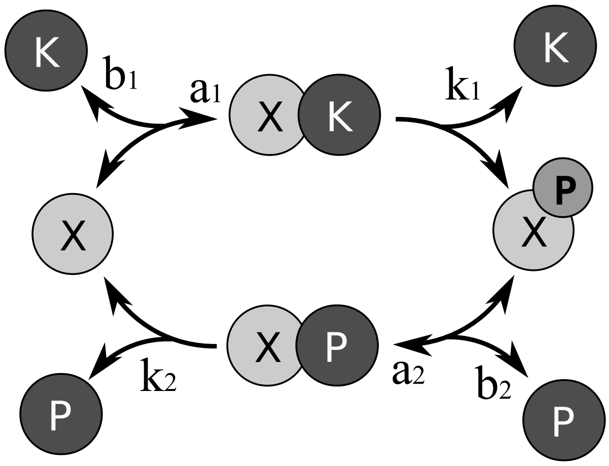

Fig. 1: **Covalent cycle wiring diagram.**

In order to center the G-K function, we multiply the independent variable for a scale factor α, $G(\alpha, x, K_1, K_2)$, where α was set in order to make the EC50 of G-K function coincides with the desired EC50 value.
