# Supplementary material for: Ultrasensitivity in signaling cascades revisited: Linking local and global ultrasensitivity estimations
Source: PLoS One. 2017 Jun 29;12(6):e0180083. doi: 10.1371/journal.pone.0180083 (PMC5491127; doi:10.1371/journal.pone.0180083)
Supplement: S2 Text — (PDF) [file pone.0180083.s002.pdf]

Supplementary Information Text S2

Ultrasensitivity on signaling cascades revisited: Linking local and global ultrasensitivity estimations

Edgar Altszyler[1], Alejandra C. Ventura[2], Alejandro Colman-Lerner[2] and Ariel Chernomoretz *[3,4]

[1]Departamento de Computación, Universidad de Buenos Aires - CONICET.
[2]Departamento de Fisiología, Biología Molecular y Celular, Facultad de Ciencias Exactas y Naturales, Universidad de Buenos Aires and Instituto de Fisiología, Biología Molecular y Neurociencias (IFIBYNE-CONICET), Ciudad Universitaria Pabellón II, C1428EHA Buenos Aires, Argentina.
[3]Departamento de Física FCEN UBA - IFIBA CONICET.
[4]Fundación Instituto Leloir.

O'Shaughnessy *et al.* model description

For the sake of completeness we included in Fig.1 the system of equations of the O'Shaughnessy's ODE models (supplementary tables S5, S7, S8 of O'Shaughnessy's *et al.* paper [**?**]).

Parameters:

$a_R = 0.9\frac{1}{\mu M.s}$; $a_M = 5\frac{1}{\mu M.s}$; $a_E = 15\frac{1}{\mu M.s}$; $k_M = k_E = 0.1\frac{1}{\mu s}$; $b_R = b_M = b_E = 0.5\frac{1}{s}$; $deg = 0.001\frac{1}{s}$; $P_R = 0.01$; $P_M = P_E = 1$

Initial conditions:

$R = 10nM$; $M = 1000nM$;$E = 1000nM$; and all other species start with zero concentration. It is worth noting that the total concentration of R, M and E, in all their forms will be held constant given the balance of protein production and degradation.

*Corresponding author: ariel@df.uba.ar

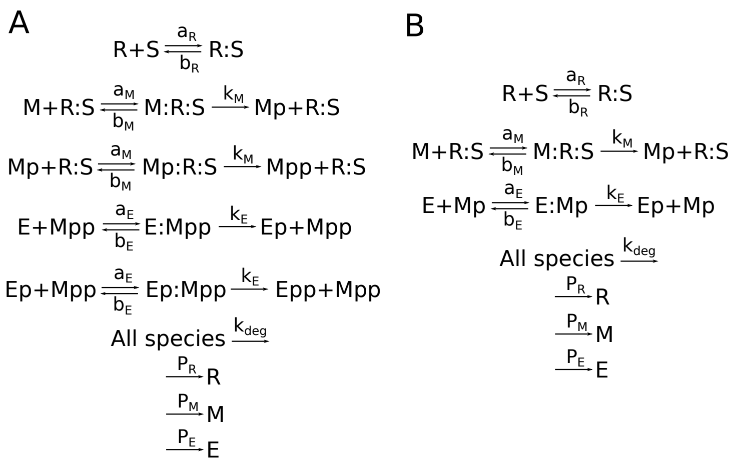

Fig. 1: **O'Shaughnessy *et al.* models reactions.**
A) Dual-step model reactions and B) single-step model reactions.
